# 3D Electrophysiological Modeling of Interstitial Fibrosis Networks and Their Role in Ventricular Arrhythmias in Non-Ischemic Cardiomyopathy

**DOI:** 10.1109/TBME.2020.2976924

**Published:** 2020-04-03

**Authors:** Gabriel Balaban, Caroline Mendonça Costa, Bradley Porter, Brian Halliday, Christopher A. Rinaldi, Sanjay Prasad, Gernot Plank, Tevfik F Ismail, Martin J. Bishop

**Affiliations:** Department of Informatics, University of Oslo 0315, Oslo, Norway, and also with the School of Biomedical Engineering and Imaging Sciences, King’s College London, London WC2R 2LS, UK; School of Biomedical Engineering and Imaging Sciences, King’s College London; National Heart and Lung Institute, Imperial College; Department of Cardiology, Guy’s and St. Thomas Hospital Trust; Cardiovascular Research Centre and Cardiovascular Magnetic Resonance Unit, Royal Brompton Hospital, and also with the National Heart and Lung Institute, Imperial College; Institute of Biophysics, Medical University of Graz; School of Biomedical Engineering and Imaging Sciences, King’s College London, and also with the Department of Cardiology, Guy’s and St. Thomas Hospital Trust; School of Biomedical Engineering and Imaging Sciences, King’s College London

**Keywords:** Arrhythmia, nonischemic, fibrosis, electrophysiology, computational model

## Abstract

**Objective:**

Interstitial fibrosis is a pathological expansion of the heart’s inter-cellular collagen matrix. It is a potential complication of nonischemic cardiomyopathy (NICM), a class of diseases involving electrical and or mechanical dysfunction of cardiac tissue not caused by atherosclerosis. Patients with NICM and interstitial fibrosis often suffer from life threatening arrhythmias, which we aim to simulate in this study.

**Methods:**

Our methodology builds on an efficient discrete finite element (DFE) method which allows for the representation of fibrosis as infinitesimal splits in a mesh. We update the DFE method with a local connectivity analysis which creates a consistent topology in the fibrosis network. This is particularly important in nonischemic disease due to the potential presence of large and contiguous fibrotic regions and therefore potentially complex fibrosis networks.

**Results:**

In experiments with an image-based model, we demonstrate that our methodology is able to simulate reentrant electrical events associated with cardiac arrhythmias. These reentries depended crucially upon sufficient fibrosis density, which was marked by conduction slowing at high pacing rates. We also created a 2D test-case which demonstrated that fibrosis topologies can modulate transient conduction block, and thereby reentrant activations.

**Conclusion:**

Ventricular arrhythmias due to interstitial fibrosis in NICM can be efficiently simulated using our methods in medical image based geometries. Furthermore, fibrosis topology modulates transient conduction block, and should be accounted for in electrophysiological simulations with interstitial fibrosis.

**Significance:**

Our study provides methodology which has the potential to predict arrhythmias and to optimize treatments non-invasively for nonischemic cardiomyopathies.

## I Introduction

NON-ISCHEMIC cardiomyopathies (NICM) are diseases of the heart muscle tissue associated with pathological ventricular dilation or hypertrophy which are not caused by atherosclerosis. A potential consequence of NICM are areas of increased fibrosis, which are detectable by late gadolinium enhanced cardiovascular magnetic resonance imaging (LGE-CMR) as areas of enhanced image intensity. Clinical studies have shown that patients with NICM and fibrosis detectable on LGE-CMR are at an increased risk of cardiac arrhythmias [1], [2]. However, the accurate risk stratification and treatment of such patients still presents a major challenge [3].

Computational modeling has emerged as a powerful tool in the study of arrythmias. Using a combination of patient-specific information from LGE-CMR, and experimental data, an in-silico representation of a patient’s heart and its pathology can be created. Such representations have been applied to determine arrhythmic vulnerability [4], and to optimize and plan treatments [5].

While computational modeling has proven useful for patients with ischemic heart disease [4], and atrial fibrillation [5]–[9], its impact on NICM has been limited so far. One reason for this is that modeling techniques and assumptions about atrial fibrillation and ischemic disease do not necessarily translate into the non-ischemic setting. In particular, the division of enhanced areas in LGE-CMR into core scar and intermediate intensity border-zone, which is commonly used in ischemic disease, is challenged in non-ischemic disease by the lack of an absolute reference from which to infer the amount of fibrosis and its electro-physiological properties [10]. This motivates the search for new modeling representations of late gadolinium enhancement (LGE) which are appropriate for non-ischemic patients with fibrosis.

In this study we model LGE as interstitial fibrosis, which has been commonly observed in histological examinations of NICM patient tissue [10], [11]. Such fibrosis results from a pathological expansion of the collagen matrix between cells, which disrupts lateral electrical connectivity and creates a potential substrate for reentrant arrhythmias [12].

We build on our previous image-guided 2D modeling methodology for interstitial fibrosis in NICM [13], based on the efficient discrete finite element (DFE) technique of Mendonça Costa *et al*. [14]. In our 2D simulations, we uncovered relationships between fibrosis density, activation delays and the likelihood of arrhythmia induction. However, important differences in electrotonic loading and electrical activation pathways through fibrosis regions between 2D and 3D motivate the need to further examine and validate these mechanisms in 3D.

Furthermore, updating the DFE method to 3D brings the important question of network topology, that is how the tissue surrounding the interstitial fibrosis is connected. This is particularly relevant to NICM in 3D, due to the potential presence of large and contiguous fibrotic areas with complex network topologies. Such large and complex 3D fibrosis networks have not been considered in the development of DFE methods to date [13]–[15], and consequently, the network topology issue has been neglected.

In the current study we present a graph theory based connectivity analysis to determine the 3D network topology, and incorporate it into a topologically consistent 3D DFE method for interstitial fibrosis. We apply the 3D DFE method in a series of image-derived cardiac tissue models, and examine the consequences on patterns of electrical activation and reentry induction. We also provide a simple 2D test-case which demonstrates that fibrosis topologies can modulate transient conduction block and hence reentry induction. This further motivates the need for a topologically consistent DFE method.

## II Methods

### A Patient MRI Dataset and 3D Geometrical Modeling

LGE-CMR images were acquired at St. Thomas Hospital from a patient with non-ischemic dilated cardiomyopathy (NIDCM) and LGE in the basal septum. A 1.5 T scanner (MAG-NETOM Aera, Siemens Healthcare, Erlangen, Germany) was used with an 18-channel body matrix coil and a 32-channel spine coil. Ten minutes after injection of 0.1 mmol/kg of gadobutrol (Gadovist, Bayer AG, Leverkusen, Germany), an inversion recovery time (TI) scout was obtained in a single mid-ventricular slice and used to determine the optimal inversion time. A respiratory navigator-gated 3D ECG-gated inversion recovery prepared sequence was acquired (resolution 1.3 × 1.3 × 2.0 mm, balanced steady-state free precession; typical TE/TR/a 1.6 ms/3.6 ms/50 degrees; GRAPPA=2) with full myocardial coverage. Ethical approval was given by the UK National Research Ethics Service (15/NS/0030), and the patient gave written informed consent in accordance with the Declaration of Helsinki.

**Fig. 1:**
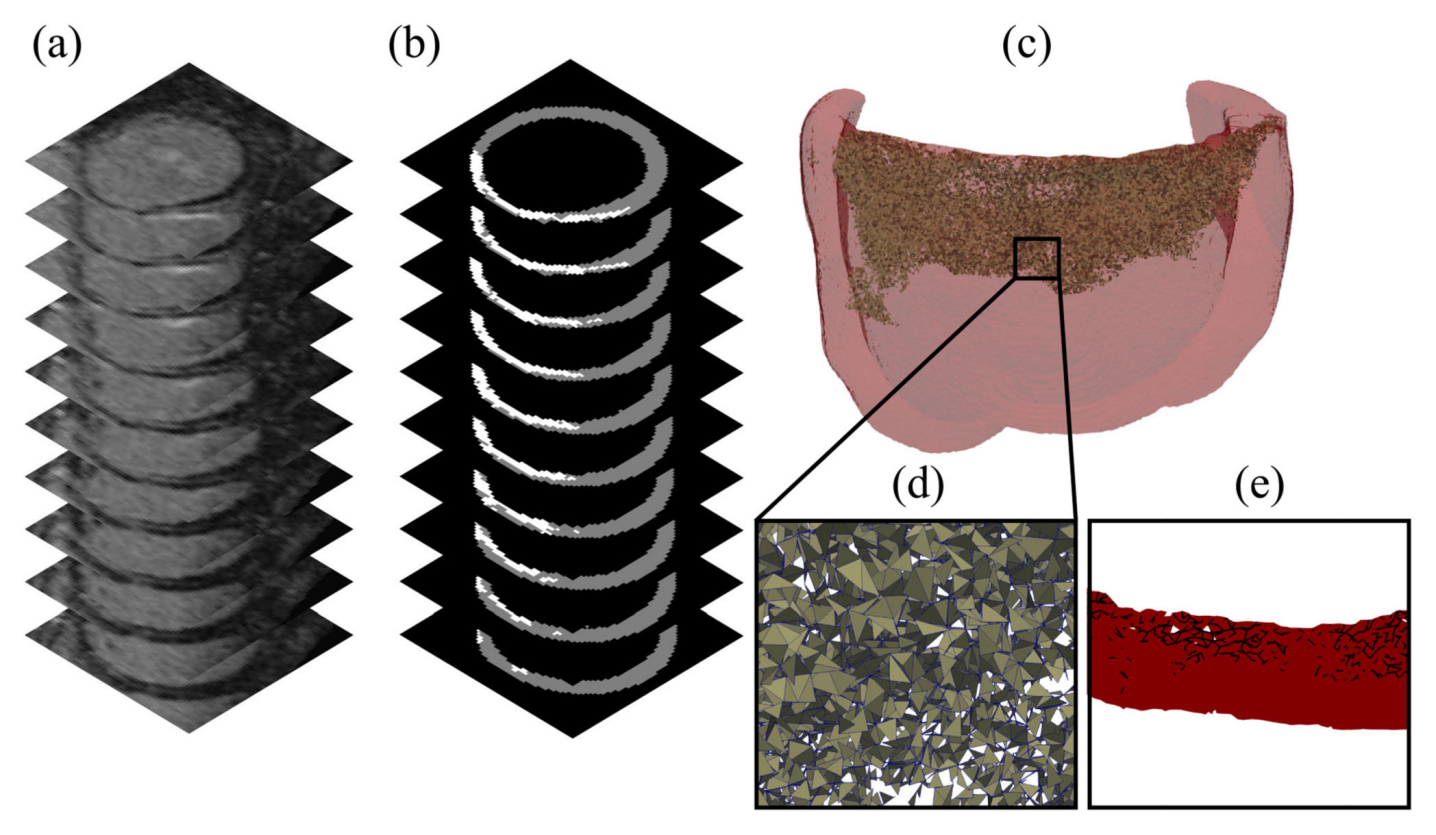
(a) Subsection of an LGE-CMR image stack of a patient left ventricle with a fibrotic zone near the valve plane. (b) Segmentation of the ventricular myocardium (gray) and fibrotic zone (white) (c) Computational mesh of all tissue within 2 cm of the fibrotic zone with split faces highlighted. (d) Close up of the split face network. (e) Projection of a section of the face network onto a plane perpendicular to the tissue walls.

The walls of the left ventricle were segmented using the medical image software Eidolon [16], and the LGE was delineated using a semi-automated full-width at half maximum technique. Tetrahedral meshes were created using CGAL [17]. For the purposes of limiting the computational expense of running detailed simulations, a reduced size mesh was created. This mesh consisted of all myocardial tissue within 2 cm of the LGE zone. The maximum edge length of the mesh was 0.25 mm within the LGE and 0.4 mm outside of the LGE. Both the mesh and example image slices are shown in Fig. 1.

In addition to the reduced mesh, a full mesh of the left ventricle was created for the determination of the local myocardial architecture. This architecture consisted of fibre, sheet and sheet normal directions, which were assigned to each element according to the rule-based method of Bayer *et al*. [18]. The relevant section of the local architecture was then transfered onto the reduced mesh via interpolation.

### B Estimation of Interstitial Fibrosis Networks From LGE-CMR

The clefts between cardiomyocytes caused by interstitial fibrosis occur on a spatial scale that is an order of magnitude below that of our LGE-CMR image resolution. We therefore required a way to estimate the location of interstitial clefts in our meshes. One approach to this problem is to randomly assign fibrosis to mesh entities [6]–[9], [13]. In our case this consisted of assigning each mesh face within the LGE a probability score between 0 and 1. This created a fibrosis probability map. Different realizations of the probability map were achieved by sampling a random number from a uniform [0,1] distribution for each mesh face within the LGE, and assigning the face to be fibrotic if the random number was larger than the probability. The selected faces formed a simulated network of interstitial fibrosis, such as the one shown in Fig. 1(c). The 0.25 mm edge length used in our network created cleavage planes comparable to experimentally observed interstitial fibrosis which separated bundles of several cardiomyocytes [19].

Given that the image intensity of LGE scales with the amount of fibrosis [20], it is reasonable to assign higher fibrosis probability scores in regions with higher intensity. We achieved this by considering the normalized fibrosis intensity

(1) $$I^{*}=\frac{I-I_{\text{ref}}}{I_{\text{max}}-I_{\text{ref}}},$$

with *I*, *I*_max_ denoting the local, and maximum LGE image intensities. The reference intensity *I*_ref_ is taken to be the mean intensity over all non-LGE voxels.

Furthermore, we assumed that clefts of interstitial fibrosis are most likely to be aligned with the local myocardial sheet architecture, due to the substantial presence of connective tissue between sheets [21]. We therefore modeled the probability of a mesh face being fibrotic as

(2) $$p=\rho_{\text{max}}(\text{cos}\theta )I^{*}$$

where *θ* is the angle between the face normal and the local myocardial sheet normal direction. The parameter *ρ*_max_ represents the global maximum fibrosis density that controls the total amount of fibrosis in the mesh. We varied *ρ*_max_ from 0.1 to 1.0 in increments of 0.1, and created 15 different fibrosis network realizations for each density, as well as a control model with no fibrosis, giving 151 different models.

### C 3D Discrete Finite Element Algorithm With Connectivity Analysis

Once we have selected a network of fibrotic faces we are left with the task of modeling the local effect of the fibrosis on electrical propagation. Ex-vivo studies of myocardial tissue with NICM [22], [23] have reported small scale conduction barriers due to fibrosis. We modeled this effect by creating no-flux boundaries along the fibrotic faces, using a nodal decoupling technique based on the method of Mendonça Costa *et al*. [14]. This method consists of creating extra copies of nodes along fibrotic edges/faces that exist in the same locations as the originals. The old and new nodes are assigned to different mesh elements, thereby creating a local discontinuity. In 2D this discontinuity is typically present along a mesh edge, whereas in 3D a mesh face is more appropriate.

We now describe how extra nodes can be created and assigned in a complex 3D network of fibrosis in such a way that a consistent topology is achieved. That is elements which are disconnected by split faces should not share nodes, giving a tight topology which does not leak current. This task is trivial when there is no branching in the network, as each face’s vertices can simply be doubled and assigned to elements on opposing sides. However, in the presence of branching, a more sophisticated procedure is required to create new nodes and assign them to the correct elements.

**Fig. 2:**
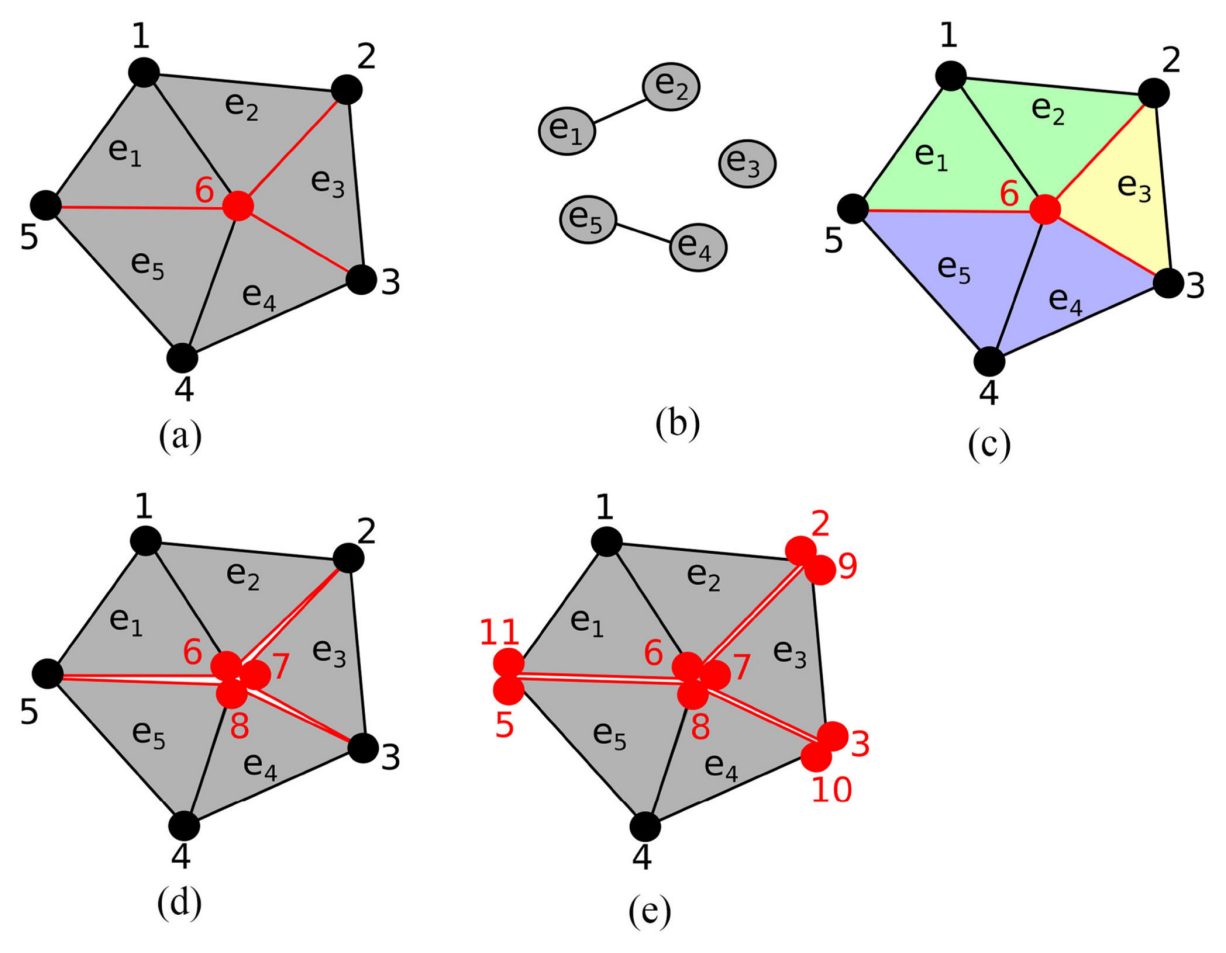
An example of local connectivity analysis to create a topologically consistent set of extra vertices. (a) The vertex 6 in the middle of the mesh is visited by the algorithm, with neighboring elements *e*_1_ – *e*_5_. The edges between *e*_2_ – *e*_3_, *e*_3_ – *e*_4_ and *e*_1_ – *e*_5_ are to be split and are marked in red. (b) The local element connectivity graph. (c) Mesh with elements colored according to their connectivity. (d) Two extra vertices are added (7, 8) and assigned to elements according to the local connectivity. (e) Later iterations of the algorithm visit nodes 2,3,5 and complete the discontinuities along the red edges.

We propose a vertex based algorithm with a local connectivity analysis to correctly assign new nodes to elements. This algorithm consists of looping over every vertex that is located on at least one split face, and performing a local connectivity analysis, as shown in Fig. 2. In this analysis a graph is built whose nodes are the mesh elements which contain the current vertex (e.g. Fig. 2(b). Two elements are connected in the graph if they share a face which is not split. The connected components of this local element graph then determine the number and assignment of the new vertices needed to achieve the local discontinuity.

**Fig. 3:**
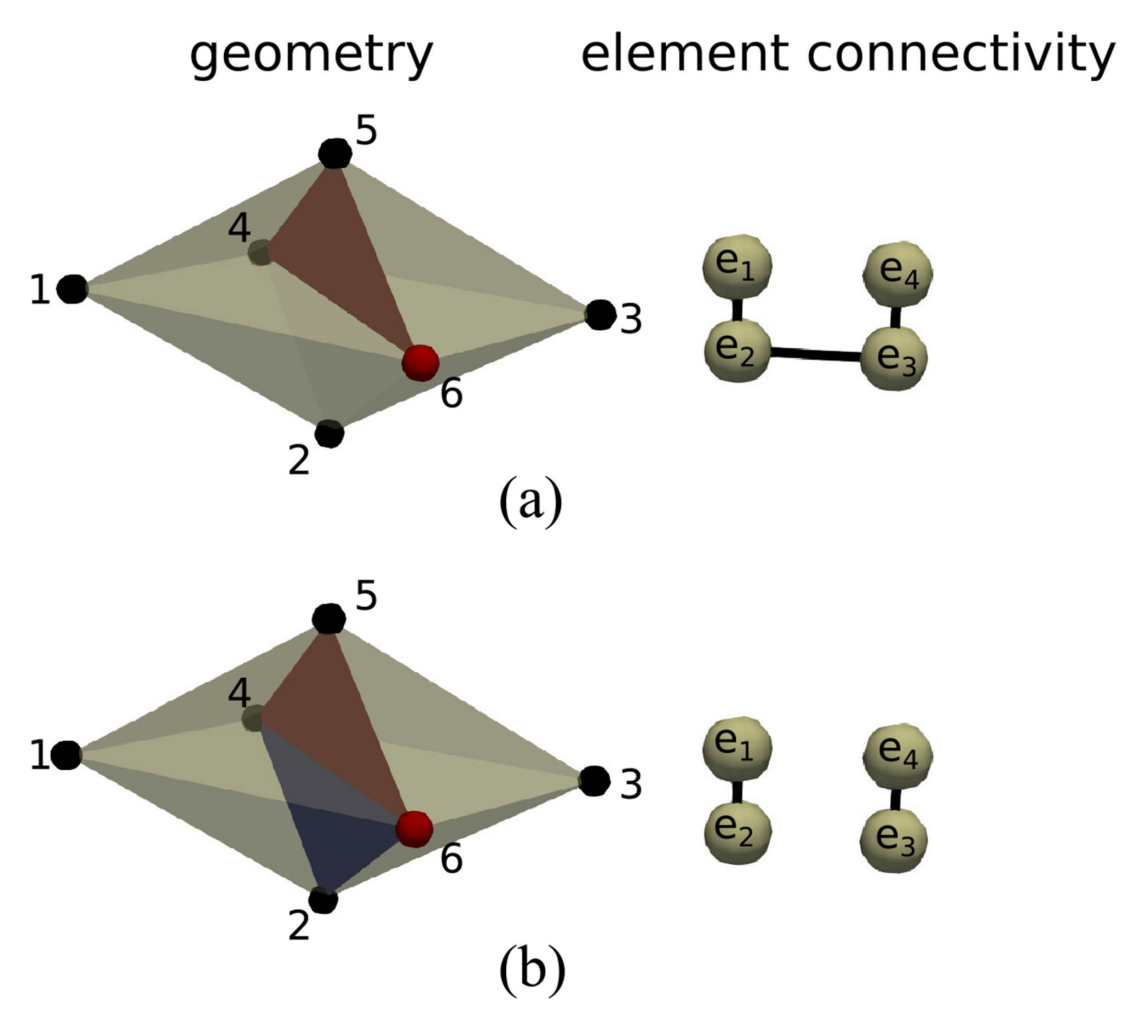
Example disconnection of a 3D connectivity graph in a tetrahedralized geometry. (a) The vertex 6 is visited by the algorithm. The split face (4, 5, 6) removes the link between *e*_1_ and *e*_4_. However, *e*_1_ and *e*_4_ are still connected via *e*_2_ and *e*_3_. (b) Face (2, 4, 6) is selected because the dot product of its normal with the normal of face (4,5,6) is minimal among all faces containing vertex 6. Elements *e*_1_ and *e*_4_ can now be isolated into two components *e*_1_ – *e*_2_ and *e*_3_ – *e*_4_. In general, several additional faces may be needed to split a 3D connectivity graph.

**Fig. 4:**
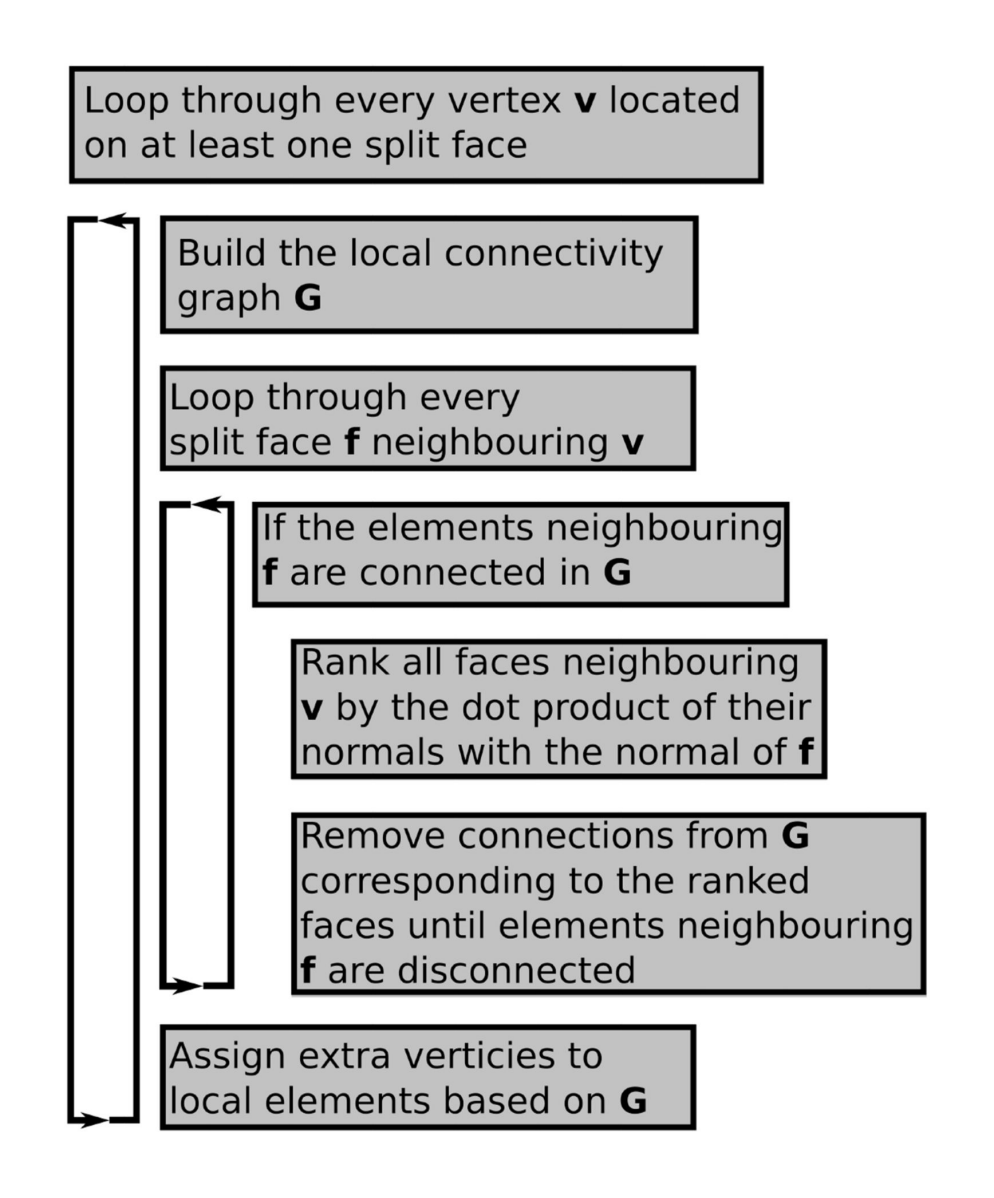
Flowchart of the 3D vertex disconnection algorithm with a modification to the local connectivity in the case that a split face does not disconnect its neighboring elements into separate groups.

It is possible that a particular splitting face does not disconnect the local connectivity graph. In 2D this can only occur if the target vertex is connected to a single split edge. Consequently, the local graph will only have a single component. In this case we can disconnect the connectivity graph along the mesh edge whose angle to the split edge is closest to 180 degrees. In 3D the situation is slightly more complicated, and we need to check that the elements on opposite sides of a split face are disconnected in the element graph. If they are not then we need a set of element faces to form a splitting plane among the local elements. This can be achieved by ranking all faces neighboring the current vertex by the dot product of the face normals with the normal of the face that we wish to disconnect. The faces with the smallest dot product can then be progressively removed from the local element connectivity until two disconnected element groups are created. An example of this procedure is given in Fig. 3. A flowchart description of the 3D nodal splitting algorithm is given in Fig. 4.

A closed surface of disconnected edges/faces may create elements which are electrically isolated. Such elements are superfluous and can be removed for computational efficiency. We achieved this by building a global element connectivity graph that is disconnected along all split faces, and then removing all connected components expect for the largest one. The mesh elements remaining in this graph are electrically connected and therefore necessary for simulation.

We have made our implementation of the mesh splitting method, along with the topology analysis, publicly available at [Online]. Available: https://github.com/GabrielBalabanResearch/lgemri_interstitial.

### D Electrophysiology Simulation

Electrical activity was simulated with the standard monodomain representation, and piecewise linear basis functions. Cellular kinetics were specified by the 2006 Ten-Tusscher model [24] of the human ventricular action potential, integrated with step size 20 *μ*s. Both monodomain and cellular kinetics were implemented in the software package CARP [25]. The primary output of interest from the monodomain model was the transmembrane potential v_m_. Activation times were recorded at the first time that v_m_ crossed 0 mV with a positive temporal derivative.

Conductivities were tuned to match experimentally observed conduction velocities [23], and accounted for the two different elements sizes (0.4 mm, 0.25 mm). In the non-LGE areas fibre conduction velocity (CVF) was 84 cm/s and transverse conduction velocity (CVT) was 23 cm/s. LGE areas were assigned reduced conductivities as in our previous study [13], that is regions in the intensity range 0–25% and 25–50% above the reference intensity, *I*_ref_, had CVT reduced by 25% and 50% respectively, with normal CVF. Regions in the intensity ranges 50–75% and 75–100% above *I*_ref_ had CVF reduced by 25% and 50% respectively, and CVT reduced by 50%.

All electrical stimuli were applied with a strength of 500 μA/cm^2^ for 2 ms. A pacing location central to the fibrosis region was chosen, based on our previous results which showed that such a location maximizes reentry incidence [13].

### E Simulated Programmed Electrical Stimulation

Simulated programmed electrical stimulation was used to test for the possibility of each 3D model to initiate an electrical reentry. The protocol consisted of a preconditioning cycle of 3 beats at 600 ms intervals, followed by up to 3 beats with dynamically determined intervals. The timing of the dynamic beats was determined by algorithmically finding the local effective refractory period using a binary search. This search began with the coupling intervals (CI) {200 ms, 450 ms} and ended when two consecutive CI were found such that the second CI initiated a new wave of activation whereas the first CI did not. A new wave was detected by the presence of any activations within 4.2 cm of the stimulus site at 110–120 ms after the stimulus initiation. After each dynamic beat 800 ms of electrical activity were simulated and a reentry was determined if any activations were present within 1 cm of the stimulus site after 300 ms.

### F 2D Test Meshes With Differing Fibrosis Topology

**Fig. 5:**
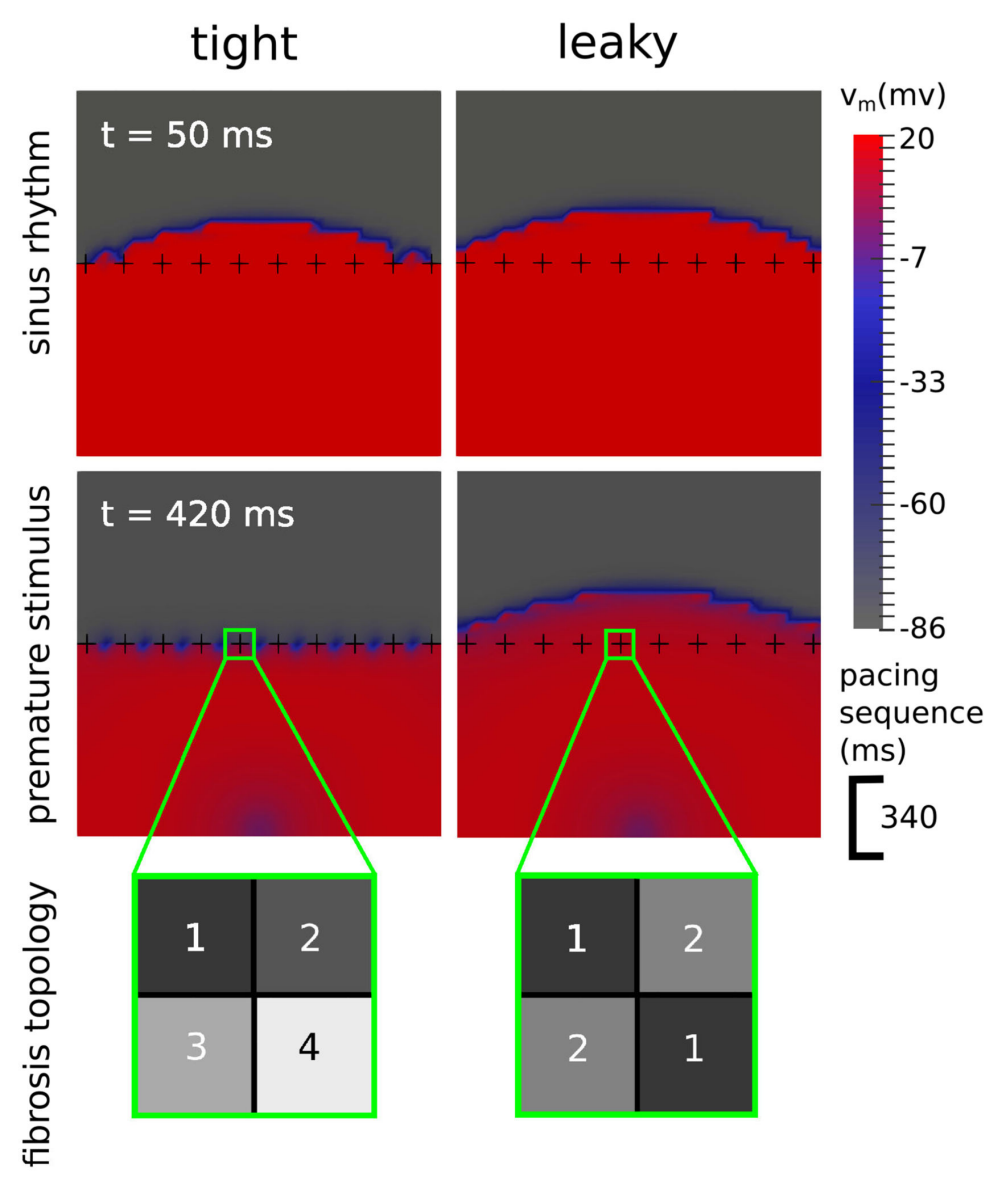
Transmembrane voltage (v_m_) maps demonstrating how the topology of a fibrosis network influences the formation of transient block. In the tight topology each fibrotic cross divides the space around it into 4 regions, whereas in the leaky topology the regions are connected diagonally, resulting in only 2 separate regions and the potential for current to leak across the fibrosis. During sinus rhythm (top row) both topologies allow an electrical wave to cross. With a premature stimulus (bottom row), 340 ms after the 1st wave, only the tight topology experiences a transient block.

To demonstrate the need for a topologically correct DFE algorithm we created a simple 2D test case consisting of 2 meshes with the same arrangement of split edges, but with differing element topologies. Each mesh had dimensions 9.5 mm × 9.5 mm, and was divided into 38 × 38 boxes, with each box consisting of two triangular elements sharing a diagonal line going from left to right. Fibrotic edges were designated in a row of 10 crosses spaced 1 element apart (see Fig. 5). In the tight topology each cross separated the surrounding elements into 4 groups, whereas in the leaky topology there were diagonal connections resulting in only 2 separate element groups. Conductivities were assigned to both meshes corresponding to an effective conduction velocity of 17 cm/s.

## III Results

### A Fibrosis Topology Modulated Transient Conduction Block

We stimulated the tight and leaky test meshes twice each, with a CI of 340 ms. This timing meant that the second wave traveled through tissue that had only partially recovered excitability, and would be therefore more susceptible to conduction block. Both stimuli were located halfway across the bottom edge.

After stimulation, the first wave crossed the row of fibrotic crosses in both meshes. The second wave however, was stopped by the tight topology but not by the leaky topology (see Fig. 5). This demonstrates that the presence of transient conduction block (a known precursor to reentry) is influenced by fibrosis topology. Using a topologically correct algorithm to model fibrosis networks is therefore an important consideration. All of the results in the next sections use a tight topology as described in Section II-C.

### B Activation Delays were Increased by Fibrosis and Faster Pacing

**Fig. 6:**
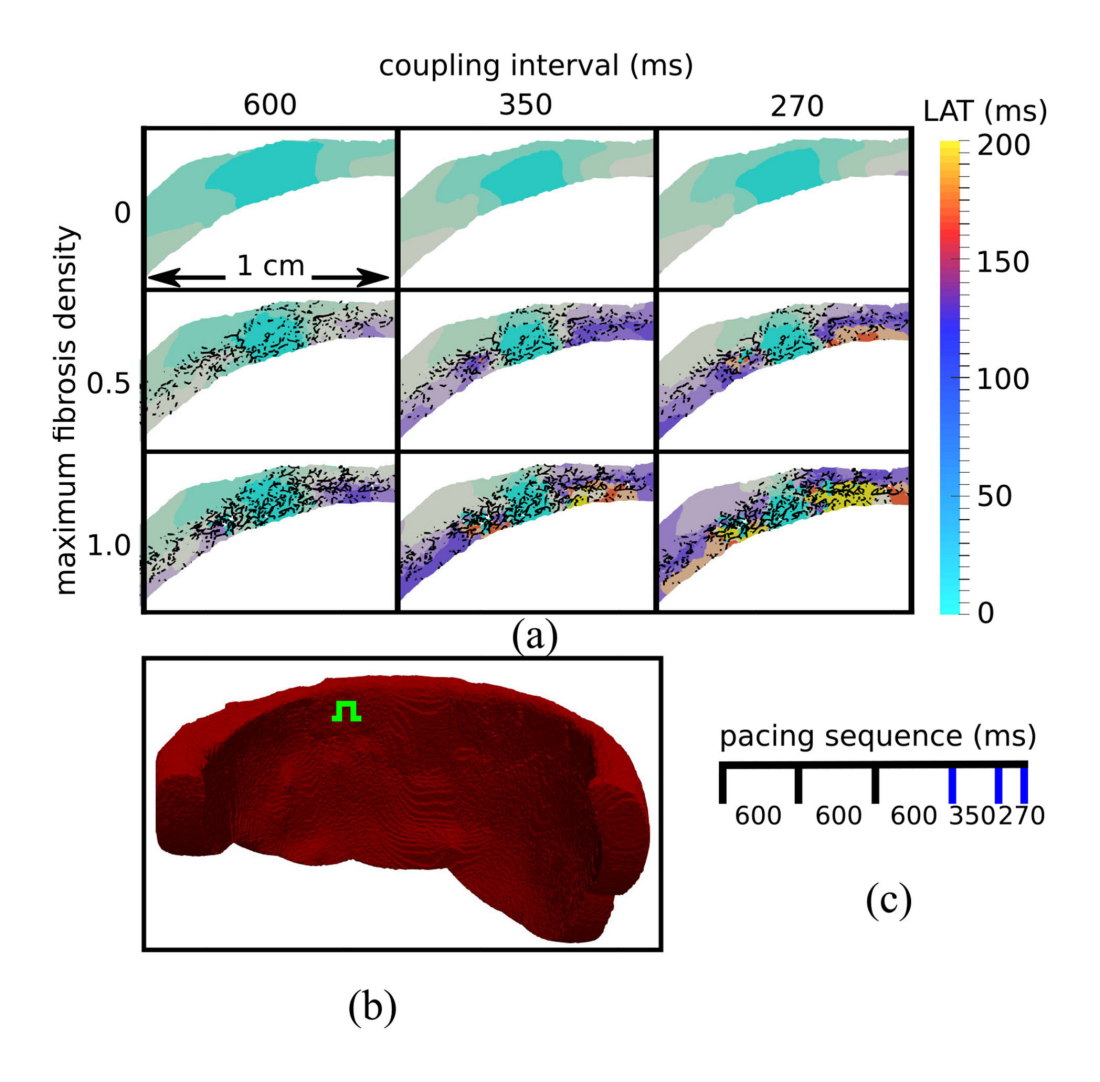
(a) Local activation time (LAT) maps in a transmural tissue slice around the stimulus location. Activation delays are larger with increased fibrosis and decreased coupling interval (Cl). The black lines represent the projection of the 3D split face network onto the LAT map plane. White areas are electrically isolated elements which were removed during preprocessing. (b) Endocardial view of the tissue geometry with the green symbol showing the location of the stimulus site. (c) Timing of stimuli with blue lines indicating stimuli whose activation maps are displayed.

We tested the effects of increased interstitial fibrosis on patterns of electrical activation in our 3-D models, using a sequence of stimuli with decreasing CI. The CI were 3 × 600 ms, 350 ms, and finally 270 ms. Activation times were measured for the final three beats, in a plane parallel to the valves and passing through the stimulus location (see Fig. 6(b). These activation maps are displayed in Fig. 6(a) for example models with fibrosis densities 1.0, 0.5 and 0 (control). The maps show that activation was progressively slowed as the CI decreased and the amount of fibrosis increased. Furthermore these effects stacked, that is both decreased CI and increased fibrosis contributed to the activation delays. Finally, we noted that activation times in the control model smoothly increased with the distance to the stimulus site. In contrast to this the activation patterns in the models with fibrosis were more irregular. This irregularity was exacerbated by faster pacing as activation pathways became more convoluted.

### C Transmural Activation Times Correlated With Reentry Incidence

**Fig. 7:**
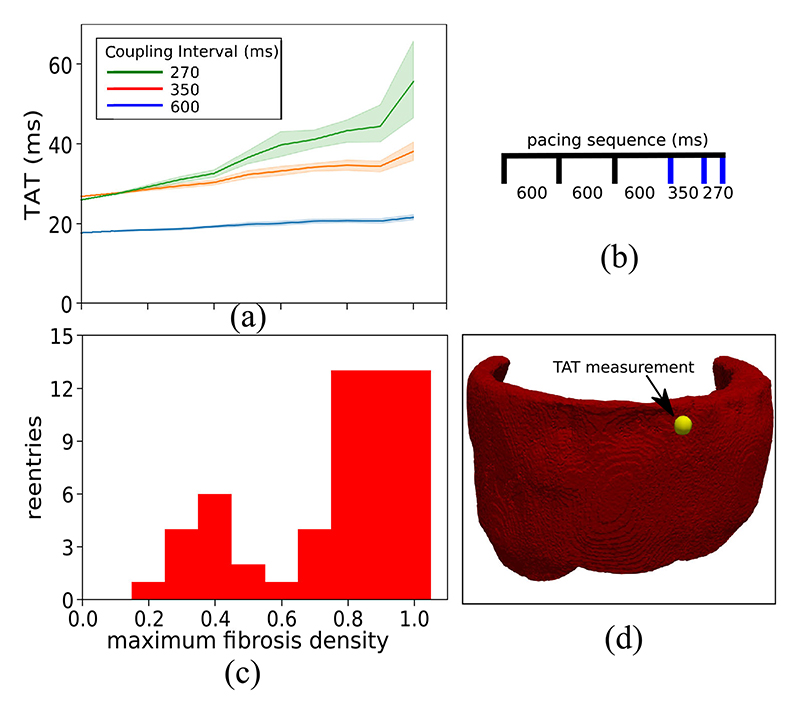
The relationship between transmural activation times (TAT) and reentries inducible by simulated programmed electrical stimulation. (a) The mean and 95% confidence region of the TAT values from 15 random fibrosis networks for each level of maximum fibrosis density. (b) Timing of stimuli used to calculate TAT scores. The blue lines indicate stimuli for which TAT was measured. (c) The number of random fibrosis networks for which reentry could be simulated at each density level. (d) Epicardial view of the ventricular geometry with TAT measurement location (yellow sphere).

Using the same stimulus location and pacing sequence as in the previous section, we measured the transmural activation time (TAT) in each model, that is the time for each wave to reach a site on the epicardium opposite to the stimulus (see Fig. 7(d). In Fig. 7(a) we display the mean and 95% confidence region of TAT for 15 models at each level of fibrosis density. We note that the mean and variance of TAT increased with the level of fibrosis, indicating a greater influence of the fibrosis network on electrical propagation. This increase was very modest at the 600 ms CI, but became more pronounced with the 350 ms and 270 ms CI. Indeed the difference in mean TAT between the models with fibrosis density 1.0 and the control model was 34 ms at the 270 ms CI.

Using the simulated programmed electrical stimulation protocol, we tested each model for the possibility of generating an electrical reentry. That is a signal that reactivated the tissue after the initial wave of activation was complete. The number of reentries that we observed for each fibrosis density level is given in Fig. 7(c). We note that there are two peaks of reentry incidence, one centered around density 0.4 and the second at densities 0.8–1.0. The first peak gave a mild reentry incidence (6/15 at density 0.4), whereas the second peak had a high reentry incidence (13/15). By comparing Fig. 7(a) and Fig. 7(c) we can see a correlation of higher TAT values with the second reentry peak.

### D The Mechanism of Reentry

We examined videos of the dynamics of the transmembrane potential v_m_ for the models that reentered to ascertain the mechanism of reentry. We noted a micro-reentry mechanism in all cases, which consistently exhibited the following phenomena: increasingly rapid pacing caused a slowing of activation and the formation of widespread conduction block in the fibrotic zone. Consequently, electrical propagation via fibrosis was forced into increasingly convoluted pathways. Finally, a reentry was initiated by one or more wavefronts meandering in the fibrosis meeting neighboring excitable tissue. Explanatory snapshots of this process, following the final extra-stimulus, are depicted in Fig. 8, a video is available in the supplement.

### E Computational Efficiency

**Fig. 9:**
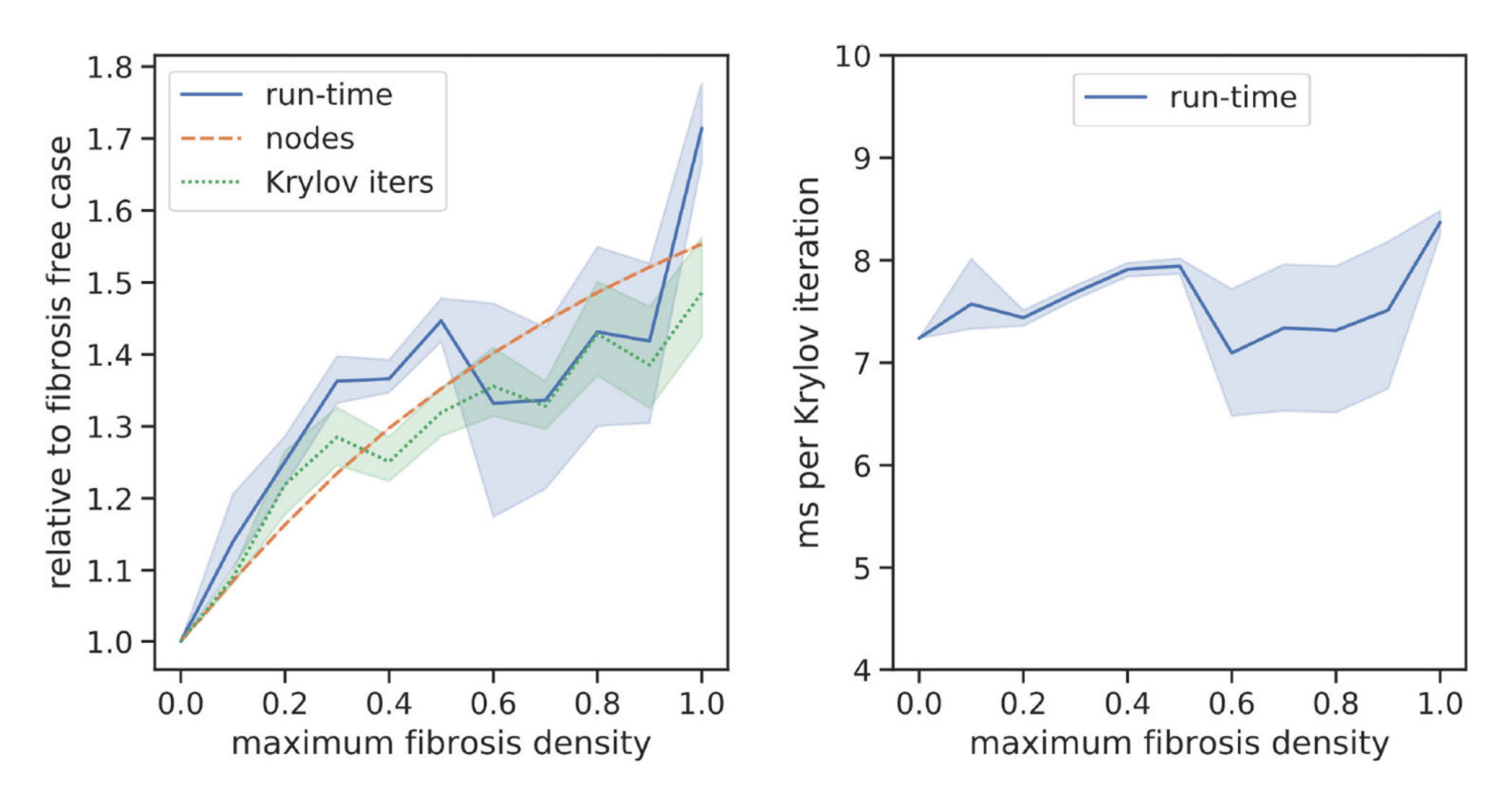
Number of mesh nodes, run-time, and Krylov iterations used to calculate the solution of the transmural activation simulations in Section III-B. The lines represent the mean over 15 simulations, whereas the shaded areas represent the 95% confidence region. Measurements in the left panel are relative to the control case, whereas the right panel shows the number of ms run-time per Krylov iteration.

We tested the computational efficiency of our DFE method by comparing the run-times and total number of Krylov iterations that we obtained from the activation delay experiments of Section III-B. The resulting data are displayed in Fig. 9. We note that increasing the fibrosis density added extra nodes to the meshes (via the fibrosis networks), and that the run-time and number of Krylov iterations scaled close to linearly with the number of nodes. There was some variation in the run-time and Krylov iterations beyond linear scaling, which we attribute to differences in the solution complexity, that is stochastic variation in the presence and size of independent wavelets caused by the fibrosis networks. The run-time per Krylov iteration was fairly stable, around 6–8 ms in all cases.

## IV Discussion

We introduced a 3D DFE method with a tight topology of interstitial fibrosis through which current cannot leak. We applied this method in an image-based model of NICM with fibrosis, and demonstrated reentrant electrical patterns in the presence of sufficient fibrosis density. Furthermore, we showed that a leaky topology may fail to simulate a transient conduction block, and hence a dangerous reentrant electrical activation. This has implications for the creation of 3D image-based models of non-ischemic fibrosis for patient risk stratification and therapy planning.

### A Fibrosis Representations

We modeled LGE as interstitial fibrosis. However, histological studies of heart tissue in NICM [10], [11] have noted a variety of other fibrosis architectures, including diffuse, patchy and compact, with multiple architectures existing in the same patient. In [13] we employed a modeling methodology for replacement fibrosis based on mesh element removal. This methodology is directly applicable in the 3D setting, and could be combined with the current interstitial model to represent mixed fibrosis architectures.

Other methodologies for representing interstitial fibrosis have been proposed [26], [27]. The method of Hooks *et al*. [26] is appropriate for small tissue samples and does not scale well to the organ level. However, the method of Trew *et al*. [27] is a comparable alternative to our method. Trew *et al*. proposed a finite-volume based formulation with disconnected element faces which automatically generates a tight fibrosis topology without requiring any connectivity analysis. However, finite element methods are very widely used for simulating cardiac electro-physiology, and the effort of adopting our method into a finite element framework is very minor. This is because all the necessary processing can be performed on the mesh before simulation, so that no changes to the finite element code are required.

### B The Importance of Fibrosis Topology

Alonso *et al*. [28] considered 2D models of cardiac tissue with a fibrotic zone with differing fibrosis representations and topologies. They found specific ranges of fibrosis density for which a reentrant activation could occur, and that these ranges depended greatly upon the type of fibrosis and its topology. Our 2D testcase with tight vs leaky topology confirms the importance of fibrosis topology. We also provide a method that creates a consistently tight topology in 3D for even the most complex networks. This method is therefore particularly applicable for future studies with multiple image-based models, which will require consistent fibrosis representations to make valid comparisons.

### C Reentry Mechanisms

The reentry mechanism in our simulations involved small activation waves propagating through narrow pathways in the fibrosis which survived long enough for the surrounding tissue to become re-excitable. Such mechanisms have been noted in previous theoretical studies on electrical percolation in excitable tissue [28], [29], in the fibrotic atria [30], after myocardial infarction [31], and in our previous 2D NICM fibrosis study [13].

Pogwizd *et al*. [32] noted focal reentrant mechanisms centered around sites with varying amounts of fibrosis in ex-planted hearts with NICM and heart failure. It is possible that microreentries could have occurred in the fibrotic zones of the Pogwizd study with a mechanism similar to what we have simulated. These would have appeared as focal activations in the sparse arrangement of electrodes that was used. Subsequent clinical studies of patients with NICM [33], [34] have observed reentrant mechanisms using catheter mapping techniques, in agreement with our simulated mechanism.

We previously noted a greater prevalence of macro and rotor reentry mechanisms rather than micro-reentries with interstitial fibrosis in our 2D study [13]. In contrast to this, we have now observed micro-reentry as the sole mechanism in our 3D models. This is most likely due to differences in fibrosis morphology between the two patients. In the current study the LGE zone was almost completely transmural. This may have prevented the formation of macro-reentries and rotors, as activation waves were forced to travel through the fibrosis rather than around it, leading to the break up of wavefronts and a micro-reentry mechanism. Further studies with greater numbers of patients are needed to confirm which reentry mechanisms are prevalent in the greater NICM population.

### D Implications for the in-vivo Identification of Arrhythmogenic Substrate

Kawara *et al*. [12] studied activation delays in tissue samples from ex-planted hearts. They concluded that fibrosis architecture greatly influences the size of activation delays, and that the largest delays are caused by patchy fibrosis with long strands rather than by diffuse fibrosis with short strands.

In the current study we show that electrical reentries are correlated with activation delays, and modulated by fibrosis density, which is in agreement with our previous 2D results [13]. This means that microstructural characteristics of fibrosis, such as its architecture and density are important factors influencing whether or not a fibrotic area is an arrhythmogenic substrate. The identification of such substrates is crucial for patient risk stratification, as well as for the targeting of catheter ablation therapy, a common treatment for cardiac arrhythmias.

Due to the limitations of current scan resolutions, the in-vivo identification of fibrosis architecture and its density is currently not possible with LGE-CMR. A potential way to circumvent this limitation is the consideration of functional electrical measurements. TAT is one such measurement and is obtainable in the ventricular septum with an opposite wall catheter technique [35]. We previously showed that abnormally high TAT values predicted reentry in 2D [13]. Our current results in Fig. 7 provide evidence for this concept in 3D, with the caveat of the first peak of reentry incidence in Fig. 7(c), which appeared at fibrosis densities that did not increase the TAT values very much. The decline in reentry incidence around density 0.6 could be due to reentrant pathways that are more likely to be closed at these densities as compared to lower fibrosis densities. Whether reentries are possible and or commonly occurring for fibrosis patterns with little to no transmural activation delay is open for future investigation.

### E Limitations

Several modeling assumptions were made in the construction of our image-based models. We assumed a completely interstitial fibrosis architecture, though a mixed architecture is more likely given histological evidence. This simplification allowed us to test our 3D DFE method in a controlled setting, thereby paving the way for its use in more complicated mixed architecture scenarios.

Experimental evidence suggests that the excitability of surviving cardiomyocytes is preserved in areas of non-ischemic fibrosis [23], [36]. This justifies the use of a model with a healthy action potential upstroke, such as the Ten-Tusscher 2006 model [24] that we use. However the experimental evidence does not rule out other kinds of pathological changes in membrane kinetics. The choice of membrane kinetics model for fibrotic areas in NICM is therefore an open question.

We also did not include any epi-endocardial differences in our cellular model [36], as the role of such differences in the acute phase of NICM (where modeling is relevant), as opposed to end stage heart failure (for which ex-vivo data are available), is currently unknown.

Our use of LGE-CMR to estimate fibrosis distributions introduces the voxel size as a potential influence on model outputs. This is because each voxel represents a block of fibrosis with similar characteristics. The fibrosis block size has been shown to influence reentry susceptibility in 2D [37], but has not yet been studied in 3D. Image voxel size may have influenced our results.

We assumed non-leaking networks of interstitial fibrosis in our LGE-CMR derived simulations, though we cannot rule out the possibility of surviving myocytes creating leaks in the network. Such a scenario could be modeled in future studies with our topology analysis by deliberately introducing leaks instead of preventing them.

Only a single image-based geometry was considered. More cases are required to generalize our results. Finally, a validation of our 3D splitting method with histological data, as in [14], [27], is lacking in the present study.

## V Conclusion

We presented a novel 3D DFE methodology which generates a consistent fibrosis topology for patients with interstitial fibrosis and NICM. Simulations in an image-derived model demonstrate that this methodology can simulate fibrosis mediated conduction delays and reentrant activations.

## Figures and Tables

**Fig. 8:**
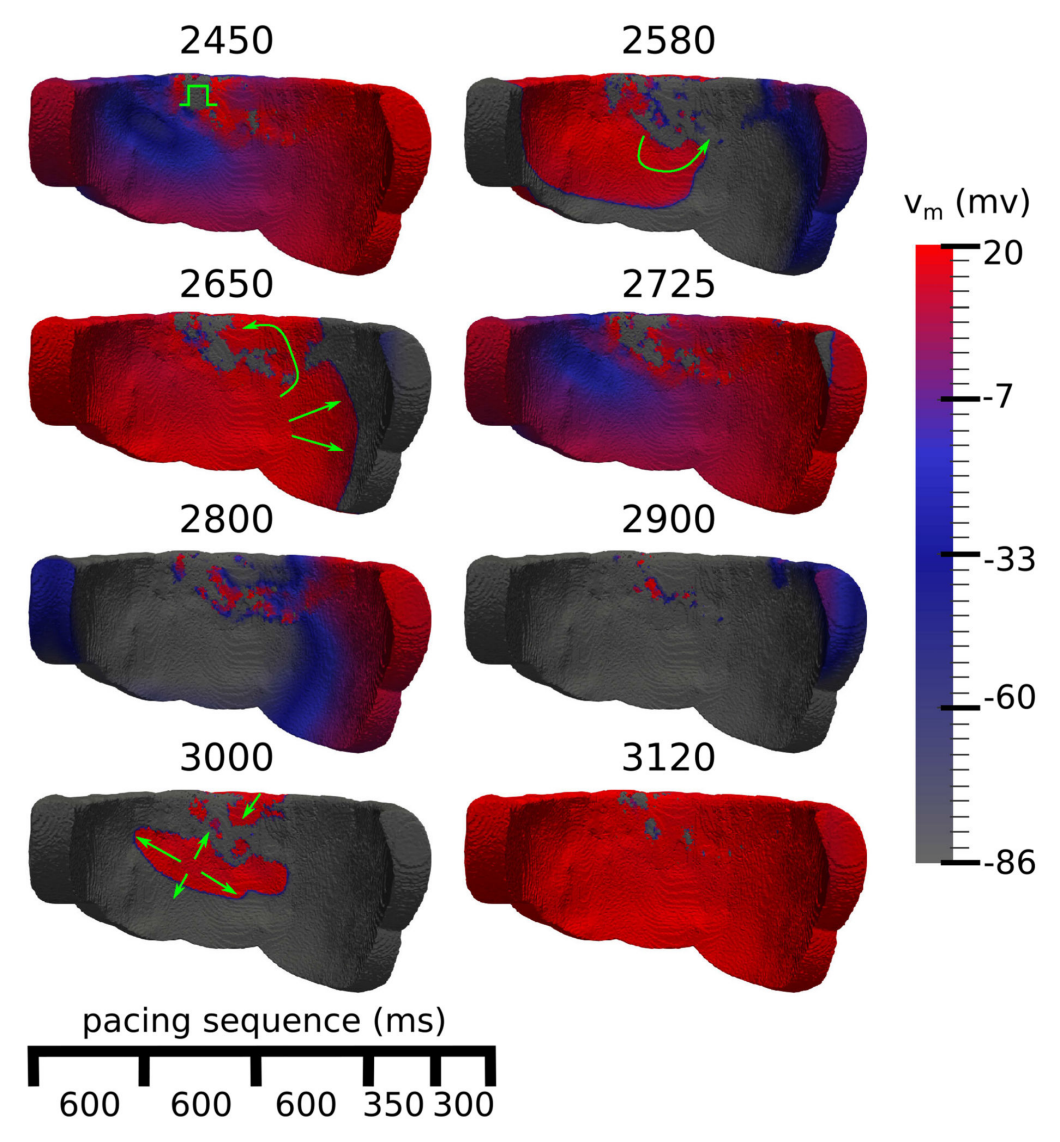
Endocardial view of transmembrane voltage (v_m_) maps after an extrastimulus that triggers an electrical reentry. The numbers at the top of each voltage map are the simulation time in ms, green arrows highlight directions of activation. 2450) The extrastimulus (green symbol) arrives into a heterogeneous repolarization landscape created by the previous stimuli. 2580, 2650) The extrastimulus spreads unevenly, first activating the tissue to the left and then later to the right. 2725, 2800) Most of the tissue repolarises. 2900) Islands of activated tissue remain in the fibrotic areas. 3000) Reentrant wavefronts emerge out of the fibrosis. 3120) Most of the tissue has been reactivated due to the reentry.
